# Brazil accounts for nearly 90% of the leprosy cases in the Americas

**DOI:** 10.1590/0037-8682-0276-2025

**Published:** 2026-02-16

**Authors:** Ana Flávia Sobral de Medeiros, Aline Lidiane Batista, Sidnei Miyoshi Sakamoto, Diogo Manuel Lopes de Paiva Cavalcanti, Renata Ferreira Magalhães

**Affiliations:** 1 Universidade Federal Rural do Semi-Árido, Departamento Ciências da Saúde, Mossoró, RN, Brasil.; 2 Universidade Estadual de Campinas, Faculdade de Ciências Médicas, Departamento de Clínica Médica, Campinas, SP, Brasil.

**Keywords:** COVID-19, Leprosy, Coinfection, Public health

## Abstract

**Background:**

Leprosy is endemic to Brazil. The Coronavirus disease 2019 (COVID-19) pandemic may have affected diagnosis and treatment.

**Methods:**

Study in Mossoró-RN (2018-2023) analyzing epidemiological data, treatment abandonment, and COVID-19 co-infection.

**Results:**

The average weekly number of cases declined during the post-pandemic period. The dropout rate was higher during the acute phase. Male sex was associated with dropout (odds ratio [OR], 6.32; *p* = 0.013). Of the 57 patients tested for COVID-19, 21 tested positive, with no clinical aggravation.

**Conclusions:**

The pandemic has affected leprosy control. Local strategies helped reduce the impact, and co-infection did not worsen the disease.

Leprosy is an infectious disease, and Brazil ranks as the country with the second highest number of new cases in the world^1^. Brazil accounts for nearly 90% of the leprosy cases in the Americas. In 2023, the incidence of leprosy in Brazil was approximately 10.68 cases per 100,000 inhabitants, with a prevalence rate of 1.21 cases per 10,000 inhabitants^1^. In this context, Rio Grande do Norte stands out for having a large number of infected patients, with Mossoró being one of the endemic cities^2^.

Since the spread of SARS-CoV-2 in 2020, there have been more than 39 million infected cases in Brazil and cumulative deaths of approximately 714000^3^. In this regard, there is concern that individuals with leprosy might be more vulnerable to developing severe forms of Coronavirus Disease 2019 (COVID-19)^4^. Therefore, we investigated the variations in the rates of leprosy diagnosis and abandonment of multidrug therapy (MDT) in the municipality of Mossoró-RN during the pre-pandemic, acute pandemic, transitional, and post-pandemic periods of COVID-19. Similarly, identifying cases of COVID-19 in patients with leprosy and leprosy reactions and their clinical outcomes were followed up in outpatient clinics in the same municipality.

For epidemiological analysis, leprosy data from 2018 to 2023 were provided by the Epidemiological Surveillance of the municipality of Mossoró-RN, which made it possible to evaluate the annual incidence and identify trends over the years. The study was approved by the Research Ethics Committee (opinion no. 4,624,975) and included patients of both sexes diagnosed with leprosy, including new cases, patients treated with MDT, type 1 and 2 leprosy reactions, and sequelae. Individuals who were unable to understand and sign the Informed Consent Form (ICF), were under 18 years of age, over 85 years of age, pregnant women, or vulnerable individuals were excluded. All the participants provided written informed consent. Sociodemographic and clinical data were collected using a structured questionnaire that covered flu-like symptoms, sociodemographic characteristics, and contact with patients with COVID-19. Capillary blood samples were collected to detect immunoglobulin M (IgM) and IgG antibodies against COVID-19 using ANVISA-certified immunochromatographic kits. Statistical analysis was performed using R® software (version 4.1.1; R Core Team), applying Fisher’s exact test for associations between categorical variables. The weekly rates were computed as the average weekly case detection and treatment default for the following calendar-defined periods: pre-pandemic (January 2018 to February 2020), acute pandemic (March 2020 to December 2021), transition (January to December 2022)., and post-pandemic (January to December 2023). Under the calendar-window approach, the mean number of new cases was 1.36/week (pre-pandemic), 1.50/week (acute), 0.94/week (transition), and 0.49/week (post-pandemic)..

An analysis of abandonment was performed using a multivariate logistic regression analysis. We performed a multivariable logistic regression for treatment default (yes/no). The covariates included sex (male vs. female), age (years), operational classification (paucibacillary vs. multibacillary), and period (pre-pandemic [reference], acute pandemic, transition, and post-pandemic). Adjusted odds ratios (aORs) with 95% confidence intervals (CIs) were calculated. Model fit was assessed using the Hosmer-Lemeshow test, and multicollinearity was evaluated using variance inflation factors (VIF). Given the low number of events and zero events in specific strata, “Transition” and “Post-pandemic” were collapsed when needed to mitigate quasi-complete separation (Table 1). Disability grade at diagnosis (0-2) was unavailable in the analytical dataset; therefore, it was not included. The limitations include the small sample size and low accuracy of serological tests during the first phase of the pandemic.

**TABLE 1:** Multivariable logistic regression for treatment default (yes/no) in leprosy cases, adjusted for sex, age, operational classification, and period; city-level dataset (Brazil, 2018-2023).

Covariate	Category (reference)	aOR	95% CI	p-value*
Sex	Male (vs Female)	1.99	0.55-445.66	-
Age	Per 1-year increase	0.98	0.95-1.00	-
Operational classification	Multibacillary vs Paucibacillary (ref PB)	0.28	0.05-1.10	-
Period	Acute vs Pre (ref Pre)	1.67	0.30-11.85	-
Period	Transition vs Pre (ref Pre)†	1.00	1.00-1.00	-
Period	Post/Transition vs Pre (ref Pre)	0.95	0.00-5.58	-

*Main inference from 95% CI (bootstrap). Asymptotic p-values suppressed due to instability with few events. † Degenerate indicator after separation treatment; maintained by transparency of the variable set. Diagnostic: Hosmer-Lemeshow χ²(8)=7,41, p=0,493; Máx. VIF = 1,22. Calibration was assessed using the Hosmer-Lemeshow test (10 deciles; χ²(8)=7.41; p=0.493), showing no evidence of lack of fit. Multicollinearity was assessed via Variance Inflation Factor (VIF), with maximum VIF=1.22, indicating negligible collinearity. Given the small number of events, the HL test may have limited power; to complement inference, 95% CIs were obtained by bootstrap. **Legend:** The model adjusted for sex (male vs female), age (years), operational classification (multibacillary vs paucibacillary, ref = PB), and period (Pre-pandemic [reference], Acute, Transition, Post/Transition*). Adjusted odds ratios (aOR) with 95% bootstrap confidence intervals (1,000 resamples) are presented. Model diagnostics: Hosmer-Lemeshow goodness-of-fit and maximum variance inflation factor (VIF). *Transition and Post-pandemic were collapsed when needed to mitigate quasi-complete separation. Disability grade at diagnosis (0-2) was not available and was therefore not included. **HL:** Hosmer-Lemeshow method; **PB:** paucibacillary.

This cohort study was conducted in a prospective, observational, and non-interventional manner in the population of the leprosy outpatient clinic located in Mossoró-RN, a municipality with 264,577 inhabitants^5^. According to the Brazilian Institute of Geography and Statistics (IBGE)^5^, Mossoró has a 0.720 Human Development Index (HDI) and is situated in the state of Rio Grande do Norte, in the northeast of Brazil. According to SINAN (Notifiable Diseases Information System), 135,226 new cases of leprosy were diagnosed in Brazil between 2018 and 2023, of which 1,226 were in the state of Rio Grande do Norte^6^, and 308 new cases were identified in the municipality of Mossoró, which characterizes the city as an endemic region. In the first phase of the study, 308 new leprosy cases registered between 2018 and 2023 were analyzed in SINAN (Notifiable Diseases Information System), considering only “mode of entry: new case” and excluding relapses, restarts after abandonment, and transfers.

The second phase of the study encompassed the most critical period of the pandemic, between August and November 2020. Between March and December 2020, 7,675,973 cases of COVID-19 were recorded in Brazil and 194,949 deaths, resulting in a mortality rate of 92.71 per 100,000 inhabitants^3^. There were 10,849 cumulative cases between March and December 2020 and 261 cumulative deaths in Mossoró, with a mortality rate of 87.77 per 100,000 inhabitants^3^.

The study began by evaluating the pre-pandemic period, which included the years 2018 and 2019, as well as the first 9 weeks of 2020, before the arrival of the pandemic, which corresponds to 113 epidemiological weeks. During this period, an average of 1.36 new cases of leprosy were diagnosed every week. The acute period ran from March 2020 to December 2021, when the pandemic peaked. This corresponds to 74 epidemiological weeks. During this period, an average of 1.50 cases/week were diagnosed. The transition period refers to the year 2022, when pandemic restrictions began to ease, and society began to adapt. This period lasted 52 epidemiological weeks. During this period, an average of 0.94 cases/week were diagnosed. Therefore, in the post-pandemic period, which began in January 2023, the data refer to 100 epidemiological weeks. The average number of new cases during this period resulted in an average of 0.49 cases/week. Thus, during the acute pandemic period, the rate of new cases was higher (1.50 cases per week) than that in subsequent periods. During the transition period, the rate of new cases fell to 0.94 cases per week, consistent with residual under-detection and the slow recovery of active case-finding. In the post-pandemic period, there was a further drop in incidence (0.49 cases/week), suggesting an accumulation of undiagnosed cases during the health crisis.

We reported the average weekly case detection rates using explicit calendar windows: pre-pandemic (January 2018 to February 2020), acute pandemic (March 2020 to December 2021), transition (January to December 2022), and post-pandemic (January to December 2023) to ensure comparability across periods of different lengths. Under this approach, the mean numbers of new cases were 1.36/week (pre-pandemic), 1.50/week (acute), 0.94/week (transition), and 0.49/week (post-pandemic). The declines during the transition and post-pandemic periods are consistent with residual under-detection and slow recovery of active case-finding following service disruption, rather than improved access.

Leprosy treatment abandonment was assessed during the study period, presenting a low proportion in total and a higher frequency in 2020 (six cases). The dropout rate was the highest in the acute pandemic period (0.08/week), followed by the pre-pandemic period (0.03), and the transition and post-pandemic periods (0.02), with no statistically significant differences. Nevertheless, multivariate multivariable analysis revealed a significant association between male gender and treatment abandonment (OR 6.32; 95% CI: 1.47-27.2; *p* = 0.013). The full coefficients and model diagnostics are listed in Table 1. These results reinforce the need for targeted follow-up strategies for men and the elderly, who are recognized as the most prone to discontinuing treatment.

However, it is essential to acknowledge the inherent limitations of this study, which may have influenced the interpretation of the results. First, reliance on secondary data from SINAN, although an official source, may involve inconsistencies or underreporting, particularly during the pandemic, when data collection may have been compromised. The low number of events in certain categories, especially treatment abandonment during specific periods, limited the statistical power of some analyses and, in some cases, introduced the risk of quasi-complete separation in the regression models, hindering the inference of robust associations. Collectively, these factors suggest that the actual impact of the pandemic may have been more profound than previously observed, underscoring the complexity of epidemiological surveillance during crises.

To verify a direct relationship between COVID-19 and leprosy, 57 patients from the leprosy outpatient clinic were tested in 2020, including 17 women (29.8%) and 40 men (70.2%) aged between 18 and 77 years. Thirty-five patients (62.7%) reported flu-like symptoms during the study period, and there was no relationship between the test results and the clinical diagnosis of COVID-19 or the medical attention sought due to respiratory complaints. Twenty-one (36.8%) participants tested positive for COVID-19. Of these, 6 were women (28.6%), and 15 were men (71.4%), with an average age of 51.6 years.

The 21 patients who tested positive did not progress to severe forms of COVID-19, nor did they show exacerbation of the underlying disease or worsening of reactions (Table 2). When analyzing the association between test results and the use of medication for leprosy reactions, it was observed that among the participants who tested positive, six were using thalidomide and eight were using prednisone. No association was found between medication use and a COVID-19 diagnosis.

**TABLE 2:** COVID-19 test results of patients with leprosy included in the study. Mossoró-RN, 2020.

Variable	COVID-19	COVID-19	**Total (*N* = 56)**	***p-*Value**	Among Positive		
	Positive (N = 21)	Negative (N = 35)			Both (N = 4)	IgM (N = 11)	IgG (N = 6)
**Paucibacillary**				0.626			
Yes	2 (9.52%)	2 (5.71%)	4 (7.14%)		0 (0.00%)	1 (9.09%)	1 (16.7%)
No	19 (90.5%)	33 (94.3%)	52 (92.9%)		4 (100%)	10 (90.9%)	5 (83.3%)
**Multibacillary**				0.369			
Yes	13 (61.9%)	16 (45.7%)	29 (51.8%)		2 (50.0%)	7 (63.6%)	4 (66.7%)
No	8 (38.1%)	19 (54.3%)	27 (48.2%)		2 (50.0%)	4 (36.4%)	2 (33.3%)
**Type 2 reaction**				0.633			
Yes	4 (19.0%)	10 (28.6%)	14 (25.0%)		2 (50.0%)	1 (9.09%)	1 (16.7%)
No	17 (81.0%)	25 (71.4%)	42 (75.0%)		2 (50.0%)	10 (90.9%)	5 (83.3%)
**Neural reaction**				0.508			
Yes	3 (14.3%)	8 (22.9%)	11 (19.6%)		1 (25.0%)	2 (18.2%)	0 (0.00%)
No	18 (85.7%)	27 (77.1%)	45 (80.4%)		3 (75.0%)	9 (81.8%)	6 (100%)

**Legend: IgM:** immunoglobulin M; **IgG:** immunoglobulin G; **MDT:** multidrug therapy; **COVID-19:** Coronavirus Disease 2019.

No association was noted between the fact that the patient tested positive for COVID-19 and the type of leprosy, treatment with multidrug therapy, or immunosuppression. In patients diagnosed with paucibacillary and multibacillary leprosy, whose treatment was based on multidrug therapy, a direct relationship existed with the diagnosis of COVID-19. Furthermore, the same was observed in patients being treated with immunosuppressants, such as thalidomide, in patients with type 2 reactions and even in cases of neural reaction treated with corticoids (*p-*values ≥ 0.05) (Table 2).

Regarding the clinical manifestations of COVID-19-positive patients, 12 cases required hospital attention due to their respiratory condition. Four of these patients were hospitalized: two in the ward, one under observation in the emergency room, and one requiring follow-up in the intensive care unit.

During the study, only one death was reported among patients with leprosy diagnosed with COVID-19, although the cause was an unconfirmed pleural effusion due to COVID-19. The other two deaths in patients who were not positive for COVID-19 were due to complications from HIV and hematological disease. There was no increased risk of mortality from COVID-19 in patients with leprosy in this sample.

Among the 21 patients who tested positive for COVID-19, 12 sought medical evaluation for respiratory symptoms, and four required hospitalization (two wards, one observation, and one ICU). Three deaths occurred in the cohort, none of which were attributable to COVID-19. There was no evidence of aggravated leprosy outcomes, increased COVID-19 severity associated with leprosy reactions, or use of thalidomide/prednisone.

During the COVID-19 pandemic, the reporting of new leprosy cases has faced significant challenges. Studies have indicated a reduction in the detection of these cases, mainly because of the overload of healthcare systems and mobility restrictions implemented to contain the virus. According to Da Paz et al.^7^, the pandemic has resulted in a substantial decrease in leprosy reports, exacerbating the existing underreporting and hindering the effective control of the disease. Although this was a global problem, we can observe that there was relative maintenance of case notification during the acute phase of the pandemic in Mossoró, RN. This was possibly due to strategies such as telecare and direct access to the leprosy outpatient clinic, as well as ongoing training on leprosy for professionals at the Basic Health Units (UBS), which allowed for continuity of patient care. Nevertheless, the decline recorded in the following periods may indicate both a lower prioritization of the active search for new cases and an overload of healthcare services in the post-pandemic period. In addition, leprosy treatment abandonment demonstrated notable variation throughout the pandemic period. According to Matos et al.^8^, there is a notable shortage of essential medicines for patients with leprosy, contributing to treatment abandonment. Furthermore, the pandemic has decreased the number of healthcare professionals available to treat leprosy, leading to inadequate treatment and support. In Mossoró, an initial increase in abandonment was observed during the peak of the pandemic in 2020; however, local strategies, such as the use of telemedicine and delivery of multidrug therapy for 3 consecutive months, helped to reduce this impact. After the pandemic, there was a gradual reduction in abandonment rates, returning to levels closer to those observed before the health crisis, suggesting that local health services had successfully adapted to the new conditions. Moreover, the association between treatment abandonment and the male sex highlights the need for specific interventions to promote therapeutic adherence and reduce the consequences of treatment discontinuation. This situation was exacerbated by profound restructuring in the organization of health services, which, before the pandemic, although already facing challenges, maintained routines of active search and contact tracing essential for diseases such as leprosy. With the emergence of COVID-19, there has been a massive reallocation of personnel and resources to address the health crisis, leading to reduced operating hours in basic health units and the suspension or severe limitation of fundamental actions such as active case-finding campaigns for new cases and the investigation of household contacts. Such deprioritization directly impacts the capacity for the early detection of leprosy, especially in paucibacillary forms, and in younger populations, who strongly depend on these proactive strategies for diagnosis. Furthermore, difficulties in accessing services and fear of viral exposure have contributed to increased treatment abandonment, with serious implications for interrupting the chain of transmission and aggravating disabilities.

Early in the pandemic, there were concerns that COVID-19 co-infection could worsen the clinical condition of patients with leprosy. However, Cerqueira et al.^9^ found no evidence that co-infection significantly worsened the condition of patients with leprosy. The data indicated that there was no notable increase in disease severity compared to that in the general population. Corroborating these findings, our study on 57 outpatients with leprosy showed that none of the 21 patients who tested positive for COVID-19 progressed to severe forms of the disease. This suggests that leprosy alone is not a risk factor for aggravation of COVID-19. In addition, our study found no significant association between the number of diagnoses and the severity of COVID-19, the use of medications for leprosy reactions, such as thalidomide and prednisone, and the diagnosis of COVID-19.

We noticed that the mortality rate was not high, with three reported deaths whose causes were not confirmed to be related to COVID-19. These findings suggest that the underlying disease and associated treatments do not increase the risk of severe COVID-19 complications. Therefore, it is essential to continue monitoring these patients, considering the socioeconomic factors that can influence exposure to the virus, as discussed by Cerqueira et al.^9^ Kahawita^10^ stated that challenges and adaptations in leprosy care in the COVID-19 era are extremely important to maintain treatment continuity and active surveillance, even in times of restriction, to prevent abandonment and ensure positive results.

These results suggest that, although patients with leprosy may have an increased risk of complications due to comorbidities, mortality directly attributable to COVID-19 may not be significantly higher in this group. The implementation of public health strategies has played a crucial role in reducing the impact of the pandemic on the treatment and follow-up of patients with leprosy.

## Data Availability

Upon request.
