# Erratum

**DOI:** 10.1590/0037-8682-0276B-2025

**Published:** 2026-03-27

**Revista da Sociedade Brasileira de Medicina Tropical/Journal of the Brazilian Society of Tropical Medicine**

Title: Brazil accounts for nearly 90% of the leprosy cases in the Americas 

In the document: Medeiros AFS, Batista AL, Sakamoto SM, Cavalcanti DMLP, Magalhães RF. Brazil accounts for nearly 90% of the leprosy cases in the Americas. Rev Soc Bras Med Trop. 2026 Feb 16;59:e0276. doi: 10.1590/0037-8682-0276-2025. PMID: 41711831; PMCID: PMC12904597

 Brazil accounts for nearly 90% of the leprosy cases in the Americas 

**Is correct**

 Impact of the Covid-19 Pandemic on Leprosy: Evidence From an Endemic City in Northeastern Brazil

